# UBQLN1 deficiency mediates telomere shortening and IPF through interacting with RPA1

**DOI:** 10.1371/journal.pgen.1010856

**Published:** 2023-07-18

**Authors:** Haoxian Zhou, Chen Xie, Yujie Xie, Yunru He, Yanlian Chen, Canfeng Zhang, Yan Zhang, Yong Zhao, Haiying Liu

**Affiliations:** 1 MOE Key Laboratory of Gene Function and Regulation, State Key Laboratory of Biocontrol, School of Life Sciences, Sun Yat-sen University, Guangzhou, China; 2 Department of Cardiology, Guangdong Provincial Cardiovascular Institute, Guangdong Provincial People’s Hospital, Guangdong Academy of Medical Sciences, Guangzhou, Guangdong, China; 3 Cardiovascular Department, The Eighth Affiliated Hospital, Sun Yat-sen University, Shenzhen, China; 4 Center for Translational Medicine, Precision Medicine Institute, The First Affiliated Hospital, Sun Yat-sen University, Guangzhou, China; Chinese Academy of Sciences, CHINA

## Abstract

Premature telomere shortening is a known factor correlated to idiopathic pulmonary fibrosis (IPF) occurrence, which is a chronic, progressive, age-related disease with high mortality. The etiology of IPF is still unknown. Here, we found that UBQLN1 plays a key role in telomere length maintenance and is potentially relevant to IPF. UBQLN1 involves in DNA replication by interacting with RPA1 and shuttling it off from the replication fork. The deficiency of UBQLN1 retains RPA1 at replication fork, hinders replication and thus causes cell cycle arrest and genome instability. Especially at telomere regions of the genome, where more endogenous replication stress exists because of G rich sequences, UBQLN1 depletion leads to rapid telomere shortening in HeLa cells. It revealed that UBQLN1 depletion also shortens telomere length at mouse lung and accelerates mouse lung fibrosis. In addition, the UBQLN1 expression level in IPF patients is downregulated and correlated to poor prognosis. Altogether, these results uncover a new role of UBQLN1 in ensuring DNA replication and maintaining telomere stability, which may shed light on IPF pathogenesis and prevention.

## Author summary

Idiopathic pulmonary fibrosis (IPF) is an age-related disease with unknown etiology but correlated with telomere length. In this study, we discovered that UBQLN1 regulates the telomere length by shuttling the RPA1 off from replication fork. The deficiency of UBQLN1 causes replication problem and telomere shortening. More importantly, knockdown the UBQLN1 at IPF mice lung cells would accelerate lung senescence and aggravate lung fibrosis. Our work demonstrates that UBQLN1 plays an important role in telomere maintenance and IPF pathogenesis.

## Introduction

Telomeres are specialized DNA-protein complexes, which are composed of tandem repeats of the TTAGGG sequence and its binding protein complex called shelterin [1]. Because of the end replication problem, telomeres shorten 10–300 bp per cell cycle and trigger cell senescence when reach a critically short length [2]. Besides the end replication problem, there are also many obstructions during telomere replication. Owing to tandem repeats of G-rich sequence, telomeric chromatin tends to form various unusual structures such as G quadruplex and heterochromatin-like structure, which are hindrances for the marching forward of the replication fork[3,4]. In addition, due to that the guanine in the DNA is more liable to be attacked by oxides, the G-rich telomeres confront more DNA damages which is also an obstacle for telomere replication [5]. Consequently, telomere length would shorten quickly if these replication stresses didn’t be solved efficiently during replication. In order to ensure that the telomeres replicate completely, human cells develop robust systems to remediate fork progression difficulties at telomeres. The cells express DNA helicases and co-factors, such as RTEL1 and BLM, to resolve the higher-order structures including G-quadruplex, triple helices, four-way junction ultrastructure that can impair fork progression[6,7]. If these genes were deficient by mutation or loss, it would cause premature telomere shortening, which then lead to accumulate senescent cell and degenerative disorders such as idiopathic pulmonary fibrosis (IPF), cardiovascular disease and bone marrow failure [8,9]. IPF is a lethal disease in middle-aged and elderly population. As telomere shortening correlates with IPF occurrence, mutations that cause telomere shortening or dysfunction are linked to IPF occurrence [10–12].

The *UBQLN1* gene encodes an ubiquitin-like protein called ubiquilin1 (also known as Plic-1), which contains an N-terminal ubiquitin-like (UBL) domain and a C-terminal ubiquitin-associated (UBA) domain [13]. The UBQLN1 protein plays multiple roles in several biological processes. It is initially reported to regulate the protein degradation through ubiquitin-proteasome system (UPS), autophagy and endoplasmic reticulum-associated degradation (ERAD) pathway [14–16]. In the recent ten years, UBQLN1 is found to participate in neurodegenerative disease development, such as Alzheimer’s disease (AD), by regulating the maturation of full-length amyloid precursor protein (APP) [17,18]. Furthermore, UBQLN1 is reported to highly express in human embryonic stem cells which possess long telomeres [19]. Nevertheless, whether and how UBQLN1 regulates telomere length maintenance and the development of other age-related diseases is largely unknown.

In this study, we found that UBQLN1 expression level is significantly downregulated in IPF lungs and patients with lower UBQLN1 display lower survival rates. We discovered that UBQLN1 ensures DNA replication efficiently and maintains telomere and genome stability by shuttling RPA1 in S phase. When UBQLN1 depleted, RPA1 retains at replication fork and hinders DNA polymerase from proceeding forward. Therefore, depletion of UBQLN1 increases replication problem and causes genome instability, especially at telomeres, the regions with much more endogenous replication stress, leading to rapid telomere shortening and accelerating lung fibrosis progress in mouse.

## Results

### UBQLN1 deficiency leads to rapid telomere shortening and genome instability

**Fig 1:**
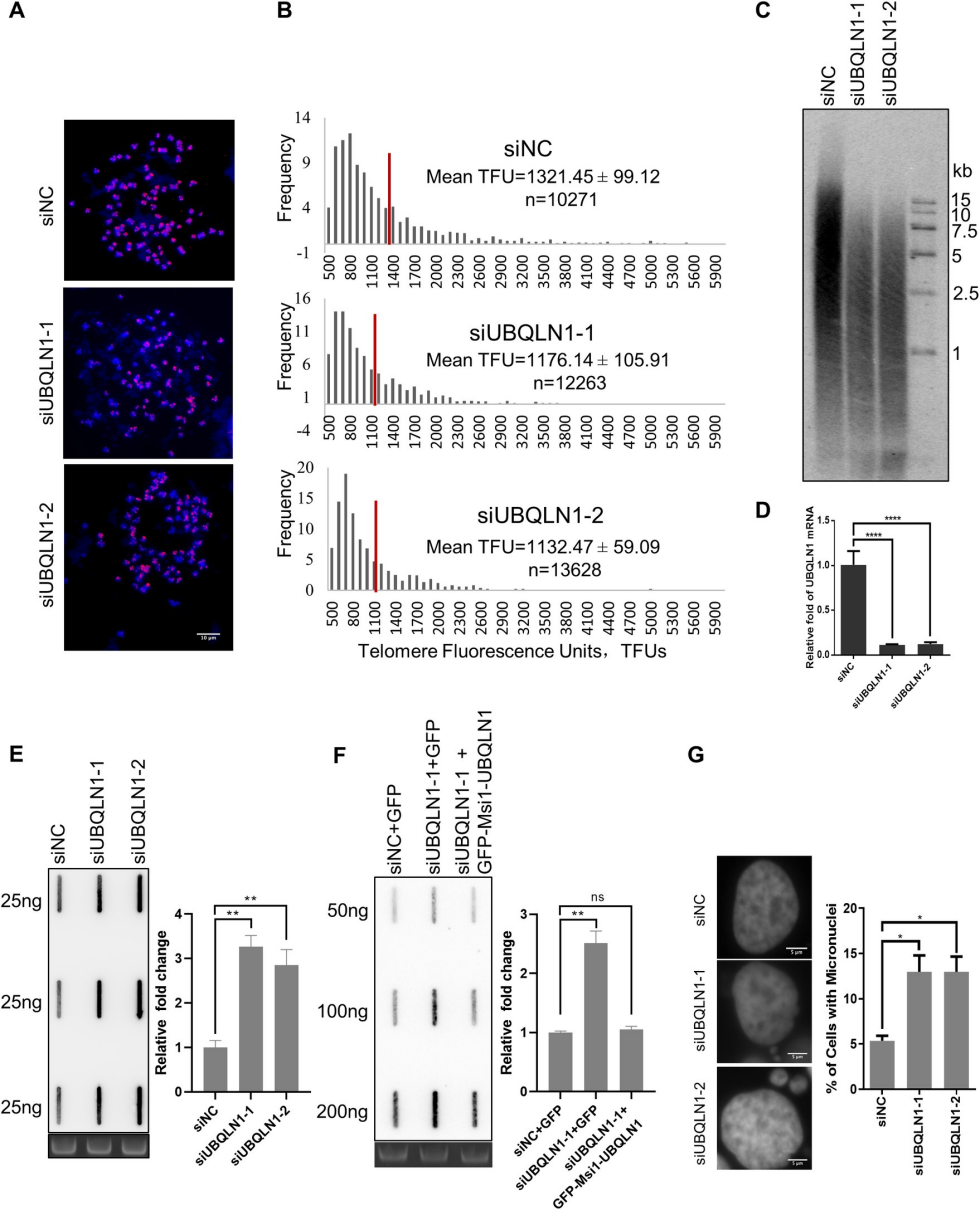
Depletion of UBQLN1 leads to genome instability. (A&B) Telomeres shortened after UBQLN1 depletion. HeLa cells were transfected with siRNAs for three days, and telomere length was detected by Q-FISH analysis (A). The telomere signal intensity was quantified (B). The numbers of quantified telomeres in Q-FISH were annotated as n. (C) Telomeres shortened after UBQLN1 depletion. HeLa cells were transfected with siRNAs twice in a week, and telomere length was detected by TRF analysis (n = 3). (D) UBQLN1 knockdown efficiency. HeLa cells were transfected as in C, total RNA were extracted and UBQLN1 mRNA was detected by RT-q PCR. (E) C-circles increased after UBQLN1 depletion in U2OS cells. Cells were transfected with siRNAs for 72h. Telomere c-circles were amplified by Φ29 DNA polymerase and detected by slot blot (DNA was plotted on nylon membrane and hybridized with telomere C-probe) (n = 3). (F) C-circles decreased after expressing synonymous UBQLN1 in 293T cells. Cells were transfected with siRNAs for 24 h and then transfected with plasmid expressing synonymous UBQLN1 for 48 h. Telomere c-circles were amplified by Φ29 DNA polymerase and detected by slot blot (n = 3). (G) Micronuclei increased after UBQLN1 depletion. HeLa cells were transfected with siRNAs for 72h, and nuclei were stained by DAPI. The micronuclei were counted. Scale bars, 5μm. All values are means ± SEM of more than three independent experiments (* P<0.05, ** P<0.01, *** P<0.001, ****P<0.0001).

It has been reported that the expression level of UBQLN1 positively correlates with telomere length by measuring the telomere length and transcriptome in a single cell simultaneously using the scT&R-seq method [19]. To explore whether UBQLN1 regulates telomere length directly, we knocked down UBQLN1 in HeLa cells and detected the telomere length by quantitative fluorescent in situ hybridization (Q-FISH) and telomere restricted fragment (TRF) assay. The results showed that telomeres shortened significantly 3 or 7 days after UBQLN1 knockdown (Fig 1A–1D). Given the rapid shortening of telomere, we proposed that telomeres suffer from much stress when UBQLN1 is depleted. Therefore, we detected the c-circle level, which is one of the hallmarks of telomere damage and instability [20,21], and found it increases in UBQLN1 depleted cells (Fig 1E). We further constructed a synonymous UBQLN1 plasmid with the siRNA targeting site mutated to restore the UBQLN1 expression in UBQLN1 depleted cells and found the c-circle recovered to normal level (Fig 1F). In addition, the 53BP1 foci, one of DNA damage markers, increase on telomeres (S1A–S1C Fig). These observations imply that telomeres are instable after UBQLN1 depletion. We also studied the effect of UBQLN1 depletion on genome stability and observed increased micronuclei (Fig 1G), implying genome instability. These results demonstrate that UBQLN1 plays an essential role in safeguarding the telomere and genome stability.

### UBQLN1 deficiency increases DNA damage and DNA replication stress

**Fig 2:**
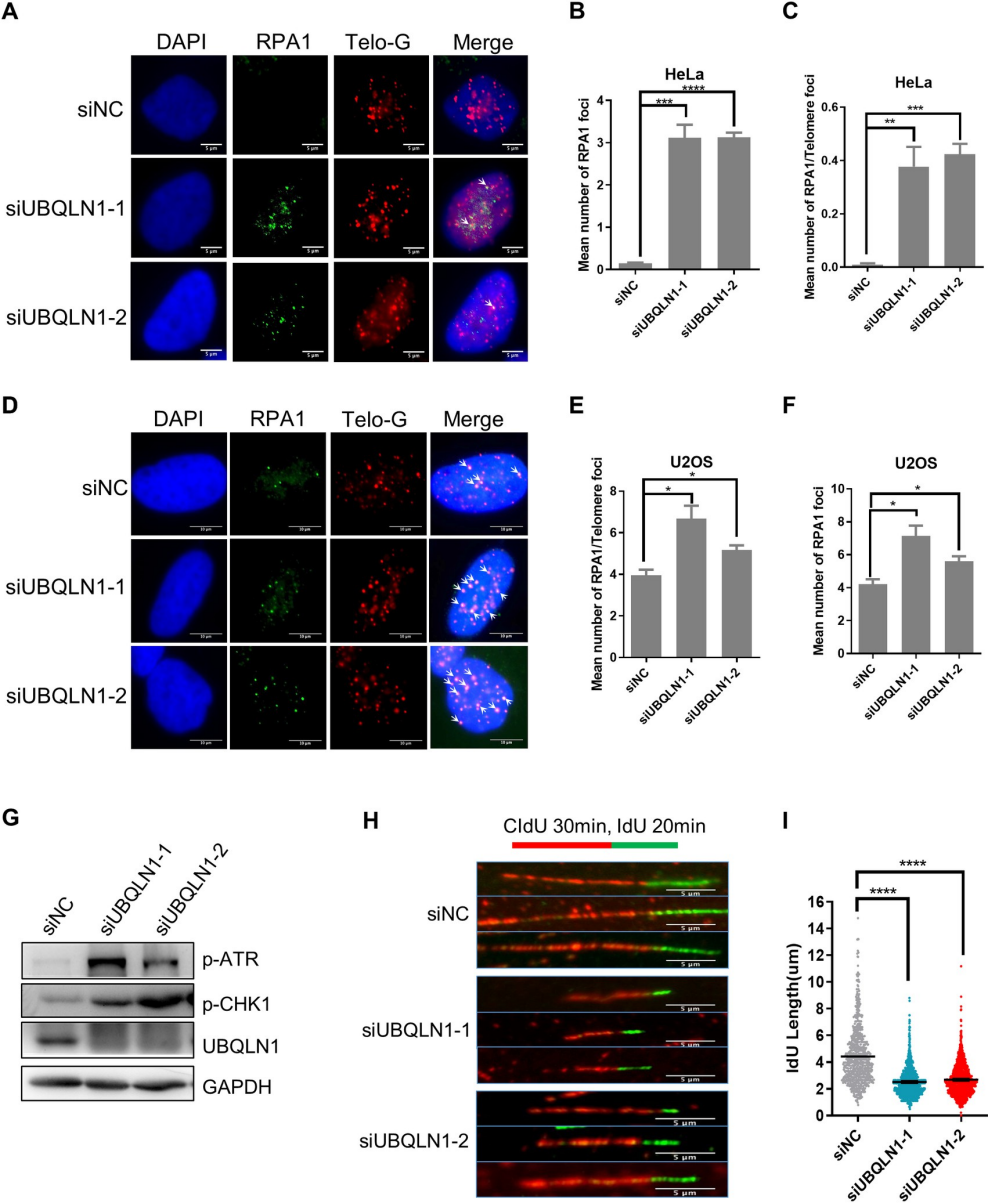
UBQLN1 is involved in DNA replication. (A) RPA1 foci increased in UBQLN1 depleted HeLa cells. Cells were transfected with indicated siRNAs for 72 h and IF was performed. Scale bars, 5μm. (B-C) Quantification of A. Cells contain more than one RPA1 foci were calculated. Total (B) or telomere localized (C) RPA1 foci were counted respectively. (D-F) Experiments in A-C were repeated in U2OS cells. (G) Protein levels in UBQLN1 knockdown cells. Immunoblot analysis of indicated proteins in HeLa cells 72 h after siRNA transfection (n = 3). (H) DNA fiber assay in UBQLN1 knockdown cells. HeLa cells were transfected with indicated siRNAs for 72 h. Cells were labeled with CldU for 30 min and subsequently with IdU for 20 min during the last 50 minutes, then the DNA fiber assay was performed. (I) Quantification of H. The length of IdU labeled DNA (green) was calculated (n ≥ 500 DNA). All values are means ± SEM of more than three independent experiments (* P<0.05, ** P<0.01, *** P<0.001, ****P<0.0001).

There are several factors that may cause telomere shorten, including the end-replication problem, telomerase activity decreases in telomerase positive cells, DNA damage, and replication defects. Given that the telomere shortening is pretty fast after UBQLN1 depletion, it couldn’t be caused by the end-replication problem, which leads to telomere loss 50 to 150 bp per cell division [22]. To confirm whether it affects telomerase activity, TRAP assay was performed after UBQLN1 depletion. The result showed that it does not affect telomerase activity (S2A Fig). In addition, the protein levels of shelterin complex do not change either (S2B Fig), suggesting the telomere structure is not affected by UBQLN1 depletion. Hence, it seems that the DNA damage and DNA replication defects become the most likely reasons, with telomere DNA damage has already been observed (Figs 1E and S1A–S1C). Furthermore, we detected the RPA1 foci on telomere. RPA1 is a single-stranded DNA (ssDNA) binding protein that stabilize ssDNA during DNA damage repair and DNA replication. We observed both total RPA1 foci and RPA1 foci on telomere are increased in HeLa, U2OS and VA13 cells (Figs 2A–2F and S3A–S3C), suggesting there are increased DNA damages or replication defects at telomere after UBQLN1 depletion. We also detected the activation of ATR signaling pathway, which responses to replication stress[23], and the results revealed that the phosphorylated ATR, phosphorylated CHK1 are increased significantly (Fig 2G), indicating that QBULN1 deficiency leads to ATR signaling pathway activation due to increased DNA damage and replication stress. Then DNA fiber experiment was performed to examine the DNA replication speed in UBQLN1 depleted cells. We labeled the DNA with CldU for 30 min and with IdU for 20 min sequentially, newly synthesized DNA was detected by immunofluorescence with corresponding antibodies. The DNA with IdU in UBQLN1 knockdown group is shorter than that in control group (Fig 2H and 2I), which means the replication forks slow down after UBQLN1 depletion. Altogether, these results demonstrate that UBQLN1 deficiency leads to increased DNA damage and DNA replication stress.

### Depletion of UBQLN1 leads to cell cycle arrest

**Fig 3:**
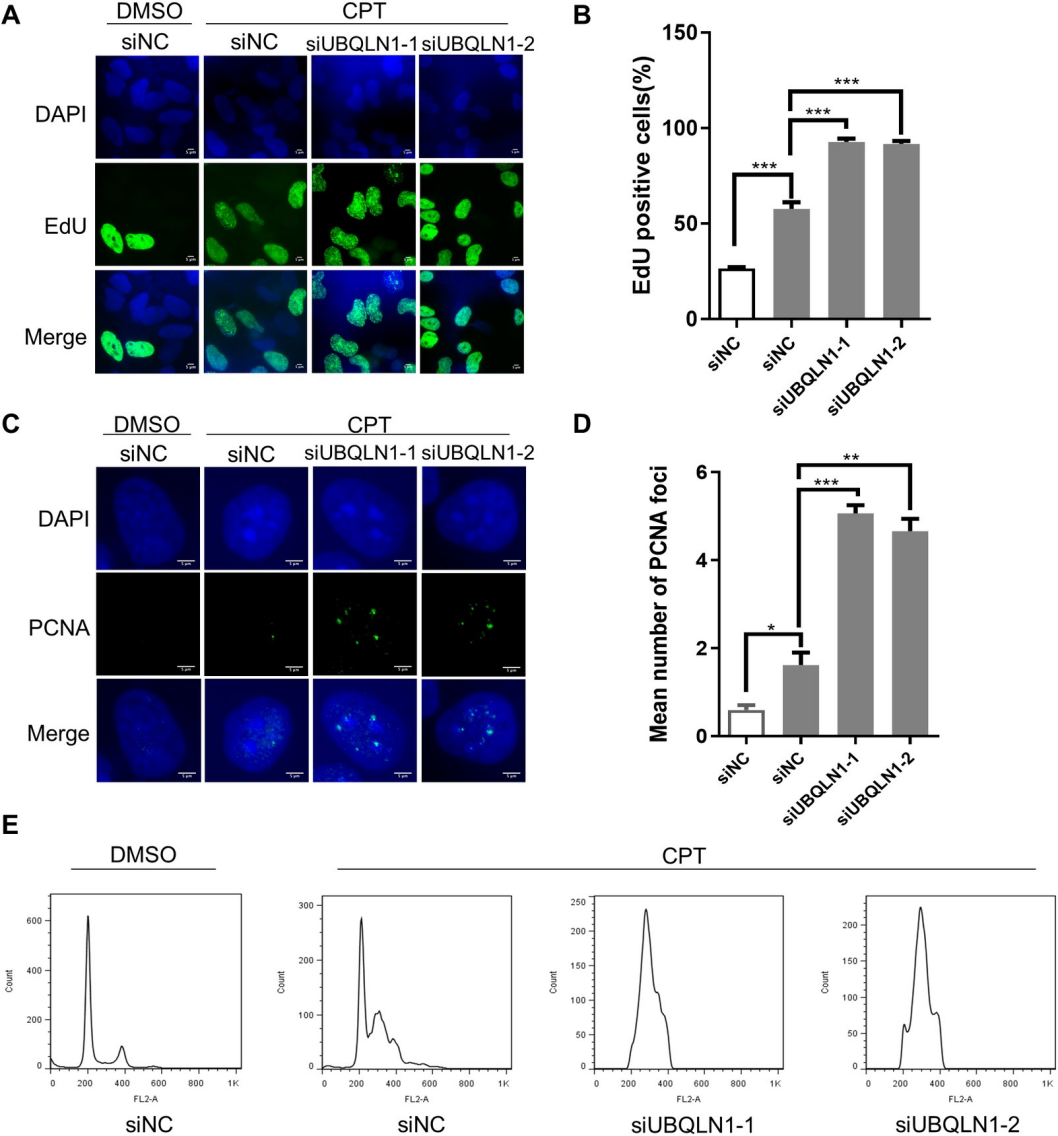
Depletion of UBQLN1 leads to cell cycle arrest. (A) UBQLN1 depletion increases EdU positive cells. HeLa cells were transfected with siRNAs for 72 h, and treated with DMSO or CPT (100 nM) during the last 12 h. Cells were treated with EdU for 15 min before detection. Scale bars, 5 μm. (B) Quantification of A. The proportion of EdU positive cells was calculated. (C) PCNA foci increased in UBQLN1 depleted HeLa cells. Cells were transfected with siRNAs and treated with CPT same as A before IF was performed. Scale bars, 5 μm. (D) Quantification of C. PCNA foci in each cell was counted, and mean number was calculated. (E) Cell cycle arrested in UBQLN1 depleted HeLa cells. Cells were transfected with siRNAs and treated with CPT same as A before flow cytometry was performed (n = 3). All values are means ± SEM of more than three independent experiments (* P<0.05, ** P<0.01, *** P<0.001, ****P<0.0001).

As complete DNA replication during S phase is the precondition for cells to pass the S/G2 checkpoint, the DNA damage and replication stress caused by UBQLN1 depletion probably delay cell cycle progression. To test this hypothesis, we firstly measured the cell viability by high content screening with all cells stained with Hoechst and dead cells with propidium iodide. It showed that the cell viability does not change after UBQLN1 depletion (S4A Fig). Then the cell proliferation was detected by CCK8 assay 72 h after UBQLN1 knockdown. The cell numbers in UBQLN1 depleted groups are significantly less than control group (S4B Fig), suggesting UBQLN1 knockdown slows down cell proliferation. Then, EdU assay was performed to test whether cells are arrested at S phase after UBQLN1 depletion. The result shows that EdU positive cells increased in UBQLN1 depleted group (S4C and S4D Fig). The camptothecin (CPT, the topoisomerase I inhibitor) treatment, which generates replication stress, boosts the EdU positive cells to about 95% in UBQLN1 depleted group (whereas 50% in control group), indicating that most cells were arrested at S phase when lacking UBQLN1 (Fig 3A and 3B). In addition, the PCNA foci also increased significantly after UBQLN1 depletion (S4E and S4F Fig; Fig 3C and 3D). PCNA is a cofactor of DNA polymerase delta, and will aggregate when cells suffer from replication problem at S phase [24]. Moreover, the flow cytometry experiment also shows that HeLa cells are arrested at S phase after UBQLN1 depletion (Fig 3E). Together, these results suggest that UBQLN1 deficient cells encounter replication problems and are arrested at S phase, especially under replication stress circumstance.

### Ubiquitination promotes interaction between UBQLN1 and RPA1

It is reported that the UBQLN1 protein binds to ubiquitinated proteins through its ubiquity-associated domain and shuttling the substrate away [25]. Moreover, many proteins will be ubiquitinated in response to DNA damage or replication stress, including RPA1 [26–28]. As we observed that UBQLN1 deficiency leads to genome instability and the RPA1 foci accumulation, it is possible that RPA1 is the substrate of UBQLN1. Therefore, we firstly determined whether RPA1 is ubiquitinated during replication stress. Plasmids expressing ubiquitin or RPA1 proteins were transfected into HeLa cells for 48 h, and the cells were treated with HU or CPT during the last 12 h. More ubiquitin signals are observed on RPA1 in cells treated with HU or CPT as compared with the untreated cells (S5A Fig), which is consistent with previous report [26].

**Fig 4:**
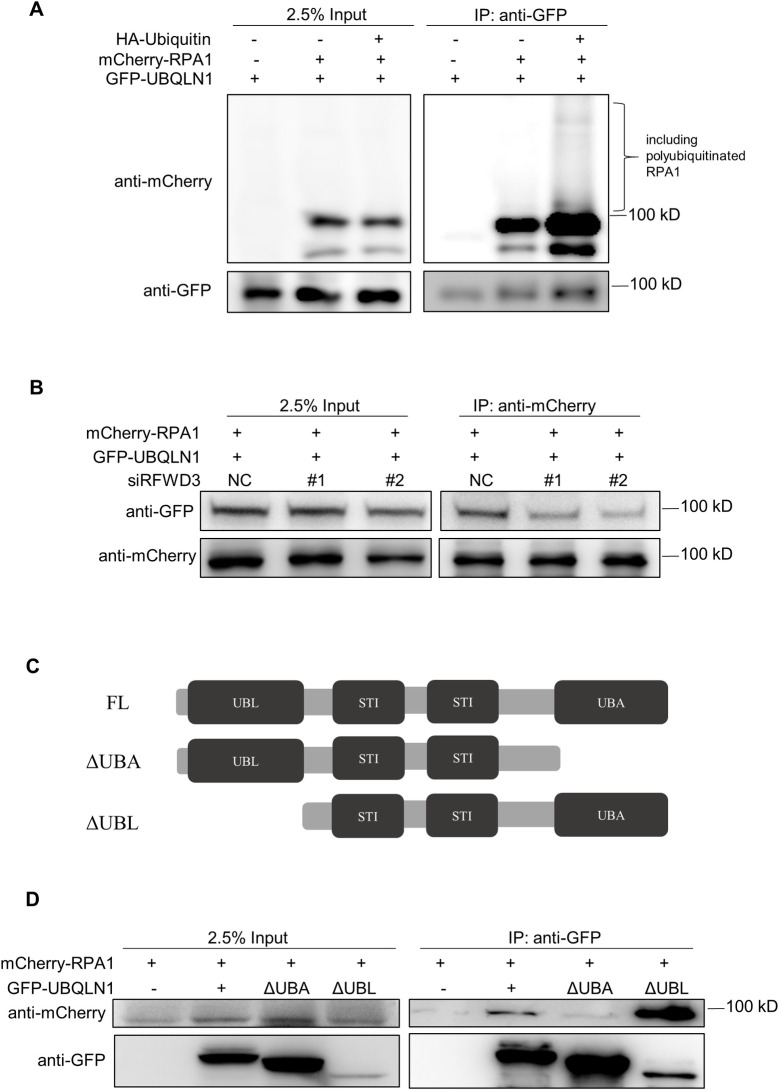
Ubiquitination promotes interaction between UBQLN1 and RPA1. (A) Ubiquitination promotes interaction between UBQLN1 and RPA1. 293T cells were transfected with plasmids expressing HA-Ubiquitin, mCherry-RPA1, GFP-UBQLN1 or vector for 72 h. Co-IP and western were performed with indicated antibodies (n = 3). (B) RFWD3 depletion weakens UBQLN1-RPA1 interaction. 293T cells were transfected with plasmids expressing mCherry-RPA1, GFP-UBQLN1 or vector for 24 h, and then transfected with siRNAs for 48h. Co-IP and western were performed with indicated antibodies (n = 3). (C) Schematic diagram showing the domains of UBQLN1. UBL: N-terminal ubiquitin-like domain; UBA: C-terminal ubiquitin-associated domain; STI: chaperone-like regions. (D) UBA domain is responsible for UBQLN1-RPA1 interaction. 293T cells were transfected with plasmids expressing full-length or truncated GFP-UBQLN1, mCherry-RPA1 or vector for 72 h. Co-IP and western were performed with indicated antibodies (n = 3).

Next, we determined whether UBQLN1 binds to the RPA1 by performing co-immunoprecipitation. We found that RPA1 was precipitated with UBQLN1, and their interaction was strengthened by expressing of ubiquitin (Fig 4A). In addition, replication stress, which resulting in protein ubiquitination, also enhanced their interaction (S5B Fig). However, under both circumstances, limited ubiquitinated RPA1 was precipitated as compared to RPA1, suggesting that UBQLN1 binds mainly to non-ubiquitinated RPA1 but the interaction is strengthened by protein ubiquitination in response to replication stress. This hypothesis was further supported by the observation that the UBQLN1-RPA1 interaction is significantly impaired when ubiquitination is reduced by E3 ligase RFWD3 depletion (Fig 4B). Next, we repeated the co-immunoprecipitation using truncated UBQLN1 proteins that lack the major domains of UBA or UBL, and the interaction is almost gone when the UBA domain (which is the ubiquitin-associated domain) but not the UBL domain is deleted (Fig 4C and 4D). Altogether, these results revealed that UBQLN1 binds to RPA1, and their interaction is facilitated in circumstance of increased ubiquitin modification such as under replication stress.

### UBQLN1 depletion retains RPA1 on ssDNA

**Fig 5:**
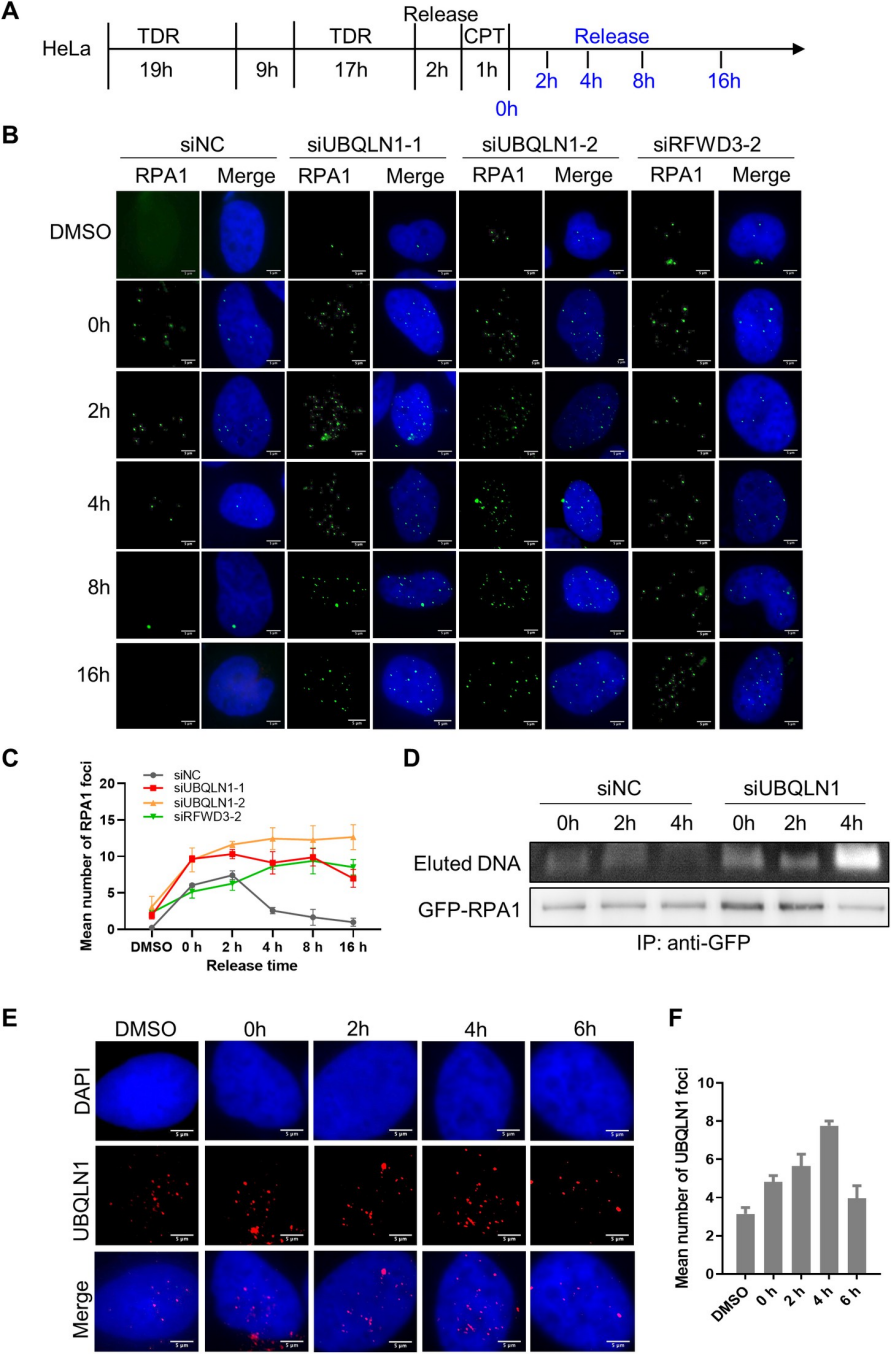
UBQLN1 regulates the departure of RPA1 from replication forks. (A) Schematic diagram of the “double thymidine” synchronization, CPT treatment and release. (B) RPA1 foci retained after UBQLN1 or RFWD3 knockdown. HeLa cells were treated as indicated in A. IF was performed with RPA1 antibody. Images were taken by Nikon Eclipse Ni-E. (C) Quantification of B. Cells contain more than one RPA1 foci were calculated (n ≥ 150 cells). All values are means ± SEM of more than three independent experiments. (D) RPA1 binds DNA persistently in UBQLN1 knockdown cells. GFP-RPA1 were stably expressed in HeLa cells, and cells were treated as indicated in A with HU treatment instead of CPT. (E) UBQLN1 foci dynamics after releasing from replication stress. HeLa cells were treated as indicated in A. IF was performed with UBQLN1 antibody at indicated time points. (F) Quantification of E. Cells contain more than one UBQLN1 foci were calculated (n ≥ 50 cells). All values are means ± SEM of more than three independent experiments.

During DNA replication, topoisomerase unfolds double-stranded DNA into single-stranded DNA, and then RPA1 binds the single-stranded DNA to prevent it from recombining into double strand. As the polymerase proceeding forward, RPA1 departures from the single-stranded DNA and thus is substituted by DNA polymerase. However, how RPA1 leaves the DNA is still unclear. To check whether UBQLN1 regulates RPA1 departing from the single-stranded DNA, HeLa cells were transfected with siRNAs, synchronized at S phase, treated with CPT and released for indicated time (Fig 5A), then RPA1 foci were detected by immunofluorescence. The results show that RPA1 foci increase from 0 to 2 hours, whereas decrease after 2 hours in control group. In contrast, the RPA1 foci are retained even after released for 16 hours in UBQLN1 depleted groups (Fig 5B and 5C). Like UBQLN1 deficiency, RFWD3 knockdown also results in RPA1 foci retention (Fig 5B and 5C).

To demonstrate whether RPA1 foci aggregates at the replication fork after deletion of UBQLN1, we performed proximity labeling in live cells using BASU, a biotin ligase that labels proximity proteins with biotin. We fused BASU to the replication marker PCNA, and performed Co-IP with Streptavidin in cells overexpressing Flag-RPA1 and HA-Ubiquitin (S6A Fig). The result showed that more RPA1 was precipitated after UBQLN1 depletion (S6B Fig), suggesting more RPA1 in the proximity of PCNA. In addition, the ChIP result revealed that in UBQLN1 depletion cells, the amount of DNA precipitated by RPA1 does not decrease as in control group for 4 h after released (Fig 5D). These results indicate that more RPA1 retained at the replication fork upon UBQLN1 depletion. Furthermore, we performed the RPA1-ChIP and found that both telomeric signals and ubiquitin were detected in the precipitation (S6C Fig), suggesting ubiquitianted RPA1 bind to telomere. The precipitated telomeric signals increased in presence of CPT, suggesting more ubiquitianted RPA1 on telomere in circumstance of replication stress (S6C Fig).

We also studied the UBQLN1 foci dynamics in HeLa cells transfected with mCherry-UBQLN1 and treated as in Fig 5A. The UBQLN1 foci increased gradually after cells released for 4 h and decreased at 6 h (Fig 5E and 5F), which is similar to the RPA1 foci dynamics with a little bit delay (Fig 5C, siNC), providing prerequisite for UBQLN1 shuttling RPA1 after it finished its job. Furthermore, we performed live cell imaging to study the dynamics of both UBQLN1 and RPA1 using cells transfected with UBQLN1-GFP and RPA1-mCherry. Cells were synchronized at S phase, treated with CPT and released as in Fig 5A. The foci with UBQLN1 and RPA1 are observed firstly in the nucleus and subsequently in the cytoplasm (S6D Fig and S1 Video). Since the UBQLN1-RPA1 foci moved out of the camera focus frequently, it cannot be tracked precisely, however, the presence of UBQLN1 and RPA1 colocalization foci in both nucleus and cytoplasm support the hypothesis that UBQLN1 shuttles RPA1 from the replication fork to the cytoplasm.

### UBQLN1 depletion retards HR repair of DSBs

**Fig 6:**
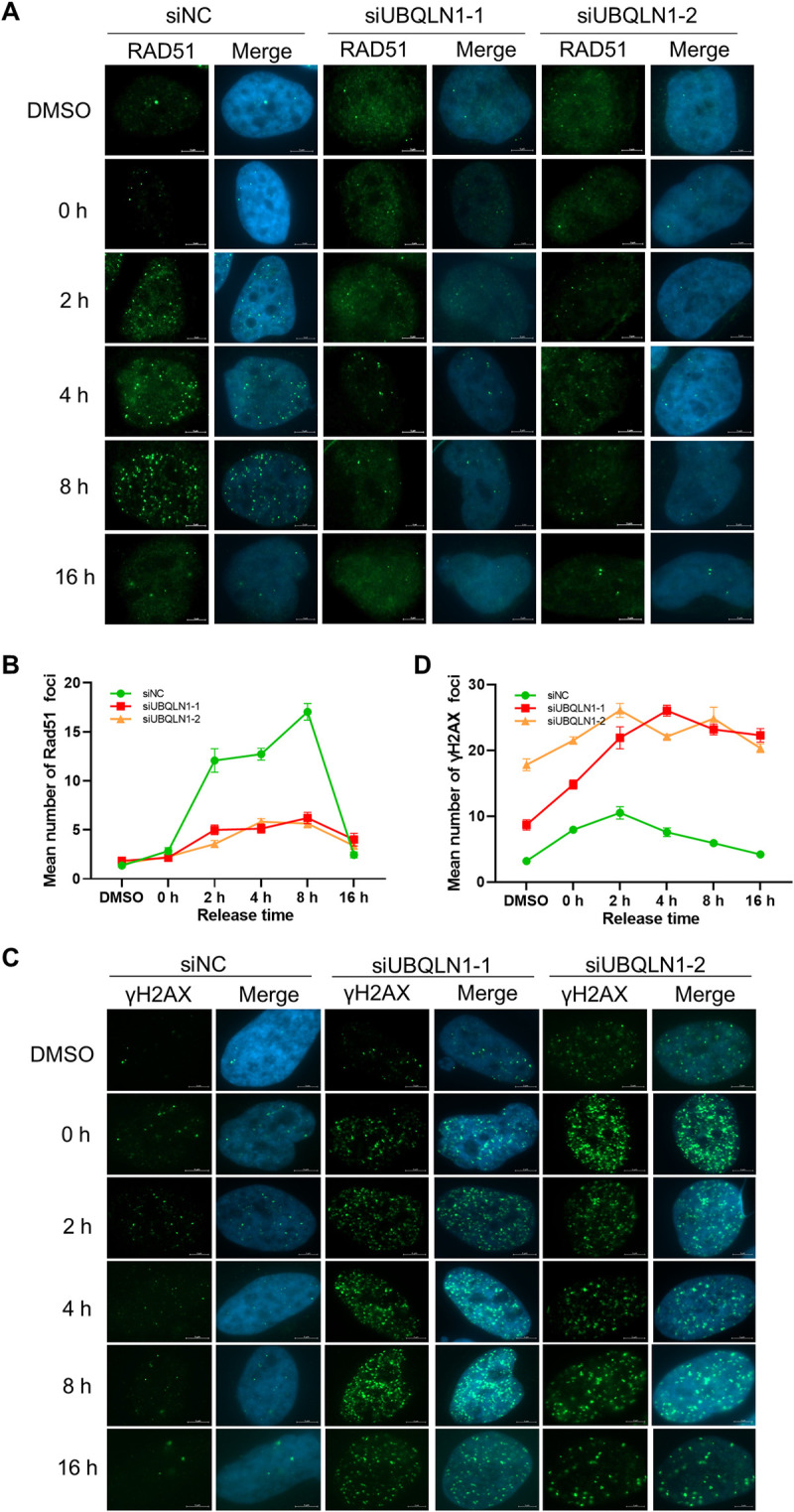
UBQLN1 depletion hinders HR repair during S phase. (A) RAD51 recruitment was hampered after UBQLN1 knockdown. HeLa cells were treated as indicated in Fig 5A. IF was performed with RAD51 antibody. Images were taken by ZEISS Axio Observer 7. (B) Quantification of A. Cells contain more than one RAD51 foci were calculated (n ≥ 50 cells). (C) γH2AX foci were accumulated after UBQLN1 knockdown. HeLa cells were treated as indicated in Fig 5A. IF was performed with γH2AX antibody. (D) Quantification of C. Cells contain more than one γH2AX foci were calculated (n ≥ 50 cells). All values are means ± SEM of more than three independent experiments.

In S phase, homologous recombination is the default mechanism for replication fork repair, during which RAD51 is supposed to substitute RPA1 on the ssDNA and then mediates the DNA strand invasion [29]. To test whether RPA1 retaining would hinder the recruitment of RAD51 to the ssDNA, we detected its dynamic in the same condition as RPA1 (Fig 5A). The results show that RAD51 foci increase from 0 to 8 h, and decrease after 8 h in control group, whereas remain at low levels after UBQLN1 depletion (Fig 6A and 6B). It suggests that RAD51 cannot be recruited properly in UBQLN1 depleted cells, which would subsequently hamper the HR repair of stalled replication fork, resulting in accumulated DNA damage. To test this hypothesis, we detected the dynamic of the DNA damage marker, γH2AX foci, and observed that it increases significantly and stays at high levels in UBQLN1 depleted cells, but keeps at low level in the control group (Fig 6C and 6D), revealing that there are more DNA damages in UBQLN1 depleted cells. Altogether, it reveals that the depletion of UBQLN1 retained RPA1 at the ssDNA, hindered replication and HR repair, resulting in accumulated DNA damage in the S phase.

### UBQLN1 deficiency aggravates idiopathic pulmonary fibrosis

**Fig 7:**
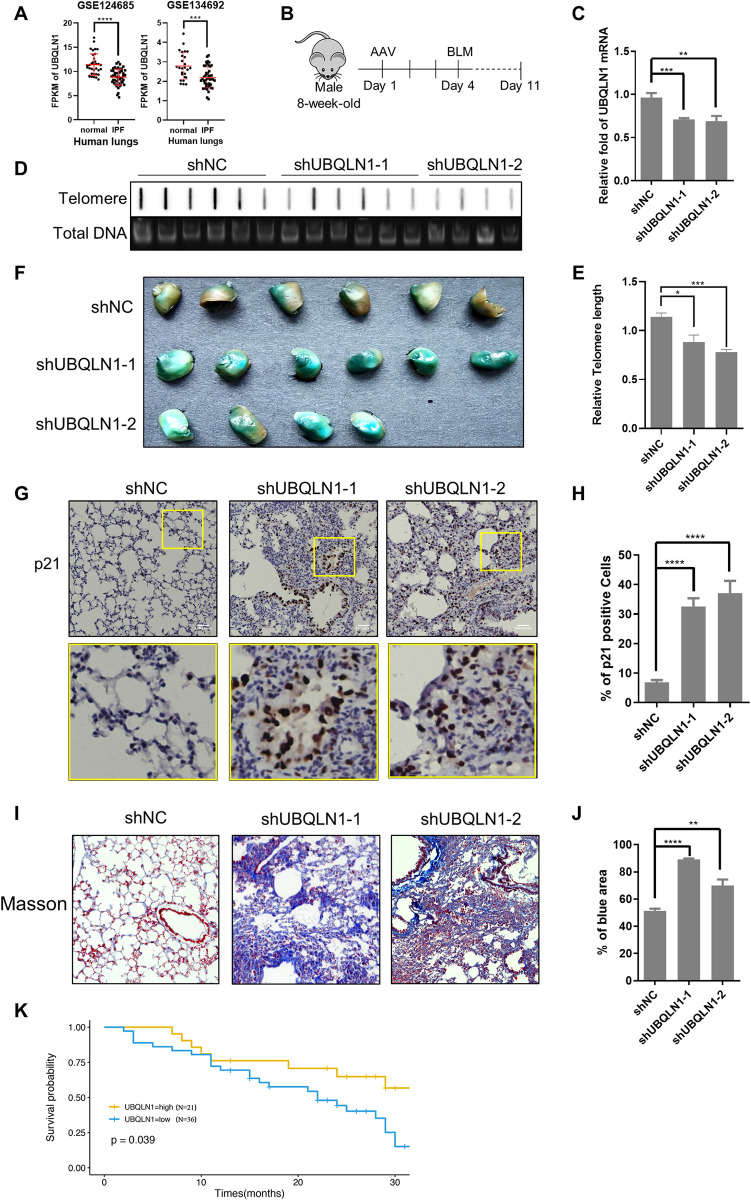
Deficiency of UBQLN1 aggravates idiopathic pulmonary fibrosis. (A) UBQLN1 transcription level decreased in human lungs of IPF patients. FPKM value of UBQLN1 gene was downloaded from RNA-seq dataset GSE124685 and GSE134692 in the GEO database. n ≥ 26 in each group. (B) Schematic diagram of mouse IPF model construction and UBQLN1 knockdown. (C) UBQLN1 knockdown efficiency in mouse model. RT-PCR analysis of UBQLN1 mRNA level in mouse lungs infected AAV carrying indicate shDNA. (D) Telomere length shortened after UBQLN1 knockdown in mouse lung. The DNA from mouse lungs in B were plotted on nylon membrane and detected by telomere C-probe. Total DNA was stained with gel-red as loading control. (E) RT-PCR analysis of Telomere length. (F) Senescent cells increased in mouse IPF lungs after UBQLN1 knockdown. Lungs from IPF mice were stained by SA-β-gal. (G) p21 protein increased in mouse IPF lungs after UBQLN1 knockdown. p21 protein in lungs from IPF mice were detected by IHC. (H) Quantification of (G). (I) Pathology of IPF after UBQLN1 knockdown. Lung tissues from mice in B were detected by Masson staining. (J) Quantification of (I). The percentage of blue area (collagenous fiber) was quantified. (K) Survival analysis of IPF patients. Data of IPF patients (GSE93606) was downloaded from GEO database. n ≥ 21 in each group. All values are means ± SEM of 4 to 6 mice. (* P<0.05, ** P<0.01, *** P<0.001, ****P<0.0001).

Idiopathic pulmonary fibrosis (IPF) is an age-related disease with unknown etiology but at least partially due to accumulated senescent cells in the tissue [30], and the patients’ survival rate is correlated with telomere length [31]. We analyzed two sets of human IPF RNA-seq data downloaded from GEO database [32,33], and found that the UBQLN1 expression level is significantly downregulated in lungs of IPF patients (Fig 7A), implying UBQLN1 deficiency potentially contributes to the IPF progress. To test this hypothesis, we constructed the bleomycin-induced pulmonary fibrosis mice, for lack of natural models of IPF and bleomycin model is the most extensively used animal model that recapitulates critical features of human IPF [34]. In this model, lung cells suffer from DNA damage, inflammation, and senescence within two weeks after bleomycin perfusion, and lung fibrosis appears in three or four weeks. We proposed that the UBQLN1 protein mainly regulates genomic stability so we chose to study the effects of UBQLN1 depletion within two weeks in bleomycin model. Before bleomycin lung perfusion, the mice were infected with AAV carrying shDNA to knock down UBQLN1 or the control sequence (Fig 7B). Mice were sacrificed at day 11 and total RNAs of the lungs were extracted to detect the UBQLN1 knockdown efficiency (Fig 7C). The telomere length of mice lung tissue was detected by slot blot and qPCR, both of which showed reduced telomere length in UBQLN1 knockdown groups (Fig 7D and 7E) and are consistent with the cell line results (Fig 1A–1D). In addition, there are more senescent cells in the UBQLN1 knockdown mice than controls, with the results showing that the lung SA-β-gal signals are stronger in the UBQLN1 knockdown mice than the control group (Fig 7F), and that the p21 expression level is significantly increased (Fig 7G and 7H). Furthermore, Masson staining was performed to study the details in the lungs, collagenous fibers (blue area) are increased whereas the number and size of pulmonary alveolus are decreased in UBQLN1 knockdown mice (Fig 7I and 7J), suggesting the IPF progress is accelerated. Finally, we performed survival analysis using downloaded clinical information of IPF patients, and found that the prognosis is worse in patients expressing low level of UBQLN1 (Fig 7K). Therefore, these data suggest that UBQLN1 deficiency aggravates IPF progress and leads to poor prognosis probably by promoting lung cell senescence.

## Discussion

Genome stability, including telomere stability, is one of the important elements that determine the cell destiny. In this study, we uncover a new mechanism that UBQLN1 interacts with RPA1 and shuttles it off from the replication fork or DNA damage site. When UBQLN1 depleted, RPA1 foci retain persistently, triggering genomic instability and leading to cellular senescence. We also found that UBQLN1 knockdown deteriorates bleomycin-induced pulmonary fibrosis progress in mice.

### Ubiquitination facilitates UBQLN1 interacts with RPA1

Ubiquitination is an important posttranslational modification during DNA damage repair and replication stress [27,28]. And RPA is an essential ssDNA binding protein complex for DNA replication [26]. In this study, we found that the ubiquitination promotes UBQLN1 interacts with RPA1 and shuttles it off the ssDNA by following evidence. First, overexpression of ubiquitin enhances the interaction between UBQLN1 and RPA1, whereas decrease RPA1 ubiquitination by knocking down RFWD3, which is the ubiquitin ligase for RPA1, attenuates the interaction (Fig 4). Second, the UBQLN1 binds to RPA1 dependent on the UBA domain, and the interaction is disrupted when the domain being depleted (Fig 4). Third, the RPA1 foci retained in the nucleus when either the UBQLN1 or the RFWD3 is depleted (Fig 5). The ubiquitination is an important modification for RPA1 to release the replication stress induced by DNA damage [26]. It is also reported that RPA must be substituted by RAD51 or DNA polymerase so that the DNA damage repair or replication can process properly [35]. Hence, our finding here raises the possibility that the ubiquitination is the signal that RPA1 it is ready to depart from the ssDNA, subsequently UBQLN1 recognizes and shuttles the RPA1, ensuring successful DNA damage repair or replication.

Shuttling off RPA1 from the replication fork by UBQLN1 is very important because RPA1 binds to ssDNA with highest affinity among the RPA members, which would block polymerase from marching forward [36]. Considering that many factors are ubiquitinated during replication [37,38], and that UBQLN1 can bind to ubiquitinated substrates, it is possible that UBQLN1 also interacts with other factors and regulates replication, but RPA1 is the one of great importance for its high affinity. Furthermore, RPA1 is ubiquitinated in response to both the replication stress and DNA damage [39,40]. Therefore, it is a question that either UBQLN1 only interacts only with RPA1 in replication at S phase, or it also interacts with RPA1 in DNA damage at other phases that retained until S phase. It remains to be answered.

### UBQLN1 depletion leads to DNA replication stress and telomere shortening

We observed that many phenotypes related to genomic instability appear in the cells when UBQLN1 is depleted, including replication fork stalling, cell cycle arrest, RPA1 foci retained, DNA damage accumulated and micronuclei increased (Figs 2, 3, 5 and 6). These evidences demonstrate that cells suffer from DNA replication stress, as RPA1 retained when UBQLN1 depleted. The replication stress probably caused by RPA1 retention in two aspects. Firstly, RPA1 needs to be replaced by DNA polymerase II, hence its retention hinders replication fork proceeding. Secondly, the RPA1 retention leads to replication stress by retarding DNA damage repair. During homologous recombination mediated DNA damage repair in the S phase, RPA1 is substituted by RAD51. The retention of RPA1 hampered RAD51 recruitment and therefore HR is retarded.

Recently, it is reported that RPA1 mutations with increased binding affinity to ssDNA lead to severe telomere shortening in inducible pluripotent stem cells [41], suggesting that proper binding or timely departure of RPA1 is important for telomere maintenance. In this study, phenotypes of telomeric instability are also observed, including telomere c-circles increased and telomere length shortened (Fig 1). It is reported that when telomeres suffer from replication stress, c-circles will be generated from telomere replication fork [42]. Hence, the increased c-circles indicate that the telomeric DNA suffers much stress after UBQLN1 depletion. Our previous study revealed that telomeres solve the stalled replication fork by looping-out mechanism which generates extrachromosomal circular structure, resulting in loss of telomeric repeats and telomere shortening [43]. Therefore, the rapid telomere shortening probably caused by looping-out of telomere replication fork suffering from stress when UBQLN1 depleted.

### UBQLN1 depletion deteriorates idiopathic pulmonary fibrosis

Shortened telomere is reported to be closely related to idiopathic pulmonary fibrosis (IPF) occurrence. Previous studies found that the leucocyte telomere lengths are shorter in patients with IPF than those with other interstitial lung diseases [44,45], and a recent mendelian randomization study using a pretty large IPF cohort (more than 3000 IPFs) demonstrated that prematurely telomere shortening is likely a cause of IPF [46]. In this study, we found a new gene, UBQLN1, mediates telomere shortening and bleomycin-induced lung fibrosis progression. The deletion of UBQLN1 leads to telomere quick shorten in both human cell line and mouse lung, further aggravates the progress of mouse lung fibrosis (Figs 1 and 7). The expression level of UBQLN1 in human IPF patients is downregulated as compared to health group (Fig 7). Furthermore, the short leucocyte telomere lengths have also been reported to be associated with poor IPF prognosis [8, 47], and we found that the IPF patients with lower level of UBQLN1 display poor prognosis (Fig 7). Consistently, we observed increased senescence cells in UBQLN1 knockdown mice lungs, which could be triggered by short telomere and genomic instability. These observations uncover a new mechanism that UBQLN1 deficiency leads to rapid telomere shortening, senescent cell accumulation and finally IPF deterioration, making it to be one of the IPF disease drivers.

## Materials and methods

### Ethics statement

Male C57 mice aged between 2–3 months were obtained from Sun Yat-Sen University laboratory animal center. Mice were bred and housed at 25 °C, 12:12 h light:dark cycle under specific pathogen-free conditions at Sun Yat-Sen University laboratory animal center. All experiments were approved by Sun Yat-Sen University Animal Care and Use Committee and were conducted in accordance with the National Institutes of Health (NIH) Guidelines for the Care and Use of Laboratory Animals.

### Cell culture

HeLa, U2OS and 293T cells were obtained from the American Type Culture Collection. Cells were cultured in DMEM (GIBCO) with 10% fetal bovine serum (GIBCO) and 100 U per ml penicillin/streptomycin (GIBCO) at 37 °C and 5% CO_2_.

### Experiments with mice

Mice were randomly assigned to experimental and control group. Mice were anesthetized via intraperitoneal injection of 10% urethane (0.2 mL/mouse), fixed on the mouse plate, and perfused with adeno-associated virus carrying shRNA (shNC, shUBQLN1-1, shUBQLN1-2) intratracheally. After recovering for two days, the bleomycin (BLM, 2.5 mg/kg body weight) was also perfused into trachea at day 4. One week after the BLM injection, mice were sacrificed. After BLM injection, 2 mice in the shUBQLN1-2 group were dead before the experiment finished and were excluded.

### Gene knockdown and overexpression

UBQLN1 and RFWD3 were deleted by siRNA using Lipofectamine RNAiMAX Transfection Reagent (Invitrogen) according to the manufacturer’s instructions. The sequences of siRNAs are as below: UBQLN1-1: 5’-GAAGAAAUCUCUAAACGUUUUUU-3’; UBQLN1-2: 5’-GUACUACUGCGCCAAAUUU-3’; RFWD3-1: 5’-GGACCUACUUGCAAACUAU-3’; RFWD3-2: 5’-AACUCCUGCACAUGACUGC-3’.

For protein overexpression, plasmids were transiently transfected into 293T cells using PEI (Yeasen), whereas HeLa cells using lipo3000 (Invitrogen) according to the manufacturer’s instructions, respectively.

### Cell cycle synchronization

Cells were synchronized using the “double thymidine” approach as previously described with a minor modification [48]. Briefly, HeLa cells were treated with thymidine (2 mM) for 19 h, washed thrice with PBS, then cultured in fresh medium for 9 h. Again, cells were treated with thymidine for 19 h followed by washing thrice before releasing for indicated time or other treatment.

### C-circle assay

C-circle assay was performed as described previously [49]. Briefly, genomic DNA was digested with the HinfI, MSPI and AfaI restriction enzymes and RNase A (NEB) at 37°C overnight. The same amount of genomic DNA was amplified by Φ29 DNA polymerase (Thermo Fisher) for 8 h at 30°C, and then subjected to slot-blot, in which DNA was plotted on nylon membrane and hybridize with telomere C-probe.

### Telomere restricted fragment (TRF) assay

The telomere length was detected by telomere restricted fragment assay as previously described [48]. A total of 5 μg genomic DNA was digested overnight at 37°C with 5 U HinfI (Thermo Fisher), 5 U Rsa1 (Thermo Fisher) and 1 μg/ml RNase A (Takara). Digested DNA samples were subjected to conventional 0.7% agarose gel in TAE buffer at 2 V/cm for 16 h at room temperature. The gel was dried at 45°C with vacuum drier, and hybridized with telomere C-probe.

### DNA fiber assay

Cells were labeled with CldU (40 μM) for 30 min and IdU (100 μM) for 20 min sequentially. Then the cells were harvested (5 × 10^5^/ml) and resuspended in cold-PBS. 3 μl of cell suspension was pipetted to the top of the aminopropylsilane-coated glass slides, and treated with 9 μl lysis buffer (0.5% SDS, 50 mM EDTA, 200 mM Tris-Cl, pH7.5) for 3 min at room temperature. And then the slides were tilted at an angle of 15° to extend the DNA. The slides were air dried overnight at room temperature and fixed in methanol/acetic acid (3:1) at 4°C for 1 h. After washing with PBS, the slides were immersed in 2.5 mM HCl for 1h to denature the DNA, and then neutralize by 0.1 M Na_3_B_4_O_7_ (pH 8.5). After washing thrice with PBST, the slides were blocked in blocking buffer (5% BSA, 0.1% Tween-20) for 20 min and then incubated with primary antibodies (anti-BrdU clone bu1/75, 1:250, Abcam, ab6326; anti-BrdU clone b44,1:50, BD, 347580) for 1.5 h. Slides were washed thrice with PBST for 5 min each time, and then incubated with the secondary antibodies for 1h. After washing thrice with PBST, slides were mounted with fluoromount. Images were captured using a Nikon microscope.

### Immunofluorescence (IF)

For immunofluorescence experiments, cells were cultured on coverslips and fixed in 4% paraformaldehyde, then permeabilized with 0.2% Triton. The coverslips were incubated sequentially with primary antibody against PCNA (1:400, ab92552, Abcam), RPA70 (1:100, sc-28304, Santa Cruz) and fluorescence-labeled second antibody (1:1000, DyLight488-conjugated anti-mouse or anti-rabbit, KPL). Coverslips were mounted with Vectashield mounting medium containing DAPI (Vector Laboratories) and visualized using fluorescence microscopy (Nikon Eclipse Ni-E or ZEISS Axio Observer7).

### EdU labeling

Cells cultured on coverslips were treated with 10 μM EdU for 15 min. Cells were washed with PBS and fixed with 4% paraformaldehyde. Cells were stained in staining buffer (1mM CuSO_4_, 10 μM FAM-azide, 10 mM sodium ascorbate in PBS). After washing with PBST, EdU-positive cells were visualized using a Nikon microscope.

### Q-FISH

Cells were treated with 0.5 μg/ml nocodazole (sigma) for 6 h to enrich cells at metaphase. Metaphase-enriched cells were hypotonic with 75 mM KCl solution, fixed with methanol/glacial acetic acid (3:1), and spread onto clean ice-cold slides. Telomeres were denatured at 85°C for 5 min and hybridized with Cy3-labeled (CCCTAA)_3_ PNA probe (Panagene). Chromosomes were stained with DAPI. Fluorescence from chromosomes and telomeres was digitally imaged on a Zeiss Axion Imager Z1 microscope. For quantitative measurement of telomere length, telomere fluorescence intensity was integrated using the TFL-TELO program.

### Western blot (WB)

Proteins were separated by SDS-PAGE and transferred to PVDF membrane. The following antibodies were incubated with membrane: anti-UBQLN1(1:1000, HPA054143, Sigma), anti-RPA70 (1:1000, sc-28304, Santa Cruz), anti-ATR pT1989 (1:1000, GTX128145, GeneTex), anti-CHK1 pS345 (1:1000, 2348T, Cell Signaling Technology), anti-Flag (1:2000, F1804, Sigma Aldrich), anti-GFP (1:2500, ab290, abcam), anti-HA (1:2000, 66006-2-Ig, Proteintech), anti-mCherry (1:1000, 26765-1-AP, Proteintech), anti-GAPDH (1:5000, 60004-1-Ig, Proteintech). And the secondary antibodies are HRP-conjugated anti-rabbit or anti-mouse (KPL, Inc).

### Co-immunoprecipitation

293T cells were transfected with indicated plasmids and lysed in RIPA buffer (1% NP-40, 0.25% Sodium Deoxycholate, 50 mM Tris-HCl (pH 7.4), 150 mM NaCl, 1mM EDTA). GFP-tagged proteins were immunoprecipitated using GFP-beads (ktsm-life, China). Coprecipitating proteins were separated by SDS–PAGE followed by western blot.

### Quantitative real-time PCR

Total RNA was extracted from cells using RNAiso Plus Reagent (9109, Takara) according to manufacturer’s instructions. 1 μg of total RNA was reverse-transcribed to cDNA using PrimeScript RT reagent Kit (RR047A, Takara). cDNA was used for real-time PCR using 2×RealStar Green Fast Mixture (ktsm-life, China). GAPDH was used as internal control for all experiments. The following primers were used for amplification: GAPDH-forward: 5’-CTGGGCTACACTGAGCACC-3’; GAPDH-reverse: 5’-AAGTGGTCGTTGAGGGCAATG-3’; UBQLN1-forward: 5’-TGCAGGTCTGAGTAGCTTGG-3’; UBQLN1-reverse: 5’-AACTGTCTCATCAGGTCAGGAT-3’; Mouse-UBQLN1-forward: 5’-TAACCCTGAAATGATGGTCCAGA-3’; Mouse-UBQLN1-reverse: 5’-TTTCTCGCAAGTTCCAGTGTT-3’.

### Chromatin immunoprecipitation (ChIP)

Cells were cross-linked by adding formaldehyde directly to culture medium to a final concentration of 1% and incubated for 10 min at 37°C, and terminated by 1.25 mM Glycine. Cells were washed twice using ice cold PBS containing protease inhibitors, then scraped and pelleted at 2000 rpm for 5 min at 4°C. Cell pellets were resuspended in 400 μL of ice-cold RIPA buffer (10 mM Tris-HCl pH 7.5, 1 mM EDTA, 0.1% sodium deoxycholate, 1% NP-40, plus protease inhibitor). Sonication was performed using the Bioruptor Plus for six times, each time 30s. Samples were then centrifuged at 13000 rpm at 4°C for 10 min, and the supernatant was transferred into a new tube. Each sample was incubated with 20 μl GFP-beads overnight at 4°C, washed once with low salt wash buffer (0.1% SDS, 1% Triton X-100, 2 mM EDTA, 20 mM Tris-HCl, pH 8.1, 150 mM NaCl), once with high salt wash buffer (0.1% SDS, 1% Triton X-100, 2 mM EDTA, 20 mM Tris-HCl, pH 8.1, 500 mM NaCl) and twice with TE buffer (10 mM Tris pH 8.0, 1mM EDTA). The protein-DNA complex was eluted twice by adding 250 μl elution buffer (1%SDS, 0.1M NaHCO_3_) at room temperature and rotated for 15 min per time. Cross-linking was reversed by adding 50 μl 3M CH_3_COONa to the complex and incubated at 65°C for 4 h. Proteins were then digested with proteinase K at 55°C for 2 h. DNA was recovered by phenol/chloroform extraction and ethanol precipitation.

### Statistics

GraphPad Prism 6 was used for statistical analysis. Results are shown as mean ± SEM and the unpaired Student’s two-tailed t-test was used to determine the statistical significance (*P< 0.05; **P< 0.01; ***P< 0.001, ****P<0.0001).

## Supporting information

S1 Fig UBQLN1 depletion increase telomere instability. (A) 53BP1 foci increased in UBQLN1 depleted HeLa cells. Cells were transfected with indicated siRNAs for 72 h and IF was performed. Scale bars, 5μm. (B-C) Quantification of (A). Cells contain more than one 53BP1 foci were calculated. Total (B) or telomere localized (C) 53BP1 foci were counted respectively. All values are means ± SEM of more than three independent experiments (* P<0.05, ** P<0.01, *** P<0.001, ****P<0.0001).
(TIF)

S2 Fig Depletion of UBQLN1 does not affect telomerase activity and shelterin expression levels. (A) TRAP assay for telomerase activity detection in UBQLN1 depleted cells. (B) Protein levels in UBQLN1 knockdown cells. Immunoblot analysis of indicated proteins in HeLa cells at 72 h after siRNA transfection.
(TIF)

S3 Fig UBQLN1 deficiency leads to DNA replication stress. (A) RPA1 foci increased in UBQLN1 depleted VA13 cells. Cells were transfected with indicated siRNAs for 72 h and IF was performed. Scale bars, 5μm. (B-C) Quantification of (A). Cells contain more than one RPA1 foci were calculated. Total (B) or telomere localized (C) RPA1 foci were counted respectively. All values are means ± SEM of more than three independent experiments (* P<0.05, ** P<0.01, *** P<0.001, ****P<0.0001).
(TIF)

S4 Fig Depletion of UBQLN1 leads to cell cycle arrest. (A) Cell viability assay after UBQLN1 knockdown. Hoechst stained all cells; PI stained death cells. (B) Relative cell number was detected by CCK8 assay. (C) UBQLN1 depletion increases EdU positive cells. HeLa cells were transfected with siRNAs for 72 h and treated with EdU for 15 min. Scale bars, 5 μm. (D) Quantification of (C). The percentage of EdU positive cells were calculated. (E) UBQLN1 depletion increases PCNA positive cells. Hela cells were transfected with siRNAs for 72 h and IF was performed. Scale bars, 5 μm. (F) Quantification of (E). The percentage of PCNA positive cells were calculated. All values are means ± SEM of more than three independent experiments (* P<0.05, ** P<0.01, *** P<0.001, ****P<0.0001).
(TIF)

S5 Fig RPA1 is ubiquitinated in response to replication stress. (A) 293T cells were transfected with plasmids expressing HA-Ubiquitin and mCherry-RPA1 or vector for 72 h, and treated with HU (1 mM), CPT (100 nM) or DMSO during the last 12 h. IP-western was performed with indicated antibodies (n = 3). (B) 293T cells were transfected with plasmids expressing HA-Ubiquitin, Flag-RPA1, GFP-UBQLN1 or vector for 72 h, and treated with DMSO, HU (1 mM) or CPT (100 nM) during the last 12 h. Co-IP and western were performed with indicated antibodies (n = 3).
(TIF)

S6 Fig UBQLN1 regulates the departure of RPA1 from replication forks. (A) Experimental schematic diagram of B. (B) RPA1 retained at replication fork after UBQLN1 knockdown. 293T cells were transfected with siRNAs and plasmids expressing Flag-RPA1, BASU-PCNA and HA-Ubiquitin for 72h, and then labeled with biotin for 6h. After crosslinking and sonication, Co-IP and western were performed with indicated antibodies. (C) RPA1 ChIP. Half of the precipitate was detected by Western blot using ubiquitin antibody, and the other half was detected by Southern blot using telomeric probe. (D) HeLa cells were transfected with GFP-RPA1 and mCherry-UBQLN1 and synchronizated at S phase as indicated in Fig 5A. The live cell was photographed using live cell imaging technology for 6 hours (n = 1).
(TIF)

S1 Video The dynamics of UBQLN1 and RPA1 interaction. HeLa cells were transfected with GFP-RPA1 and mCherry-UBQLN1 for 24 h, and then synchronizated at S phase as indicated in Fig 5A. The live cell was photographed using confocal fluorescence microscope live cell imaging technology for 6 hours (take one photo every three minutes).
(MP4)
