# Supplementary figures and images for: UBQLN1 deficiency mediates telomere shortening and IPF through interacting with RPA1
Source: PLoS Genet. 2023 Jul 18;19(7):e1010856. doi: 10.1371/journal.pgen.1010856 (PMC10381042; doi:10.1371/journal.pgen.1010856)

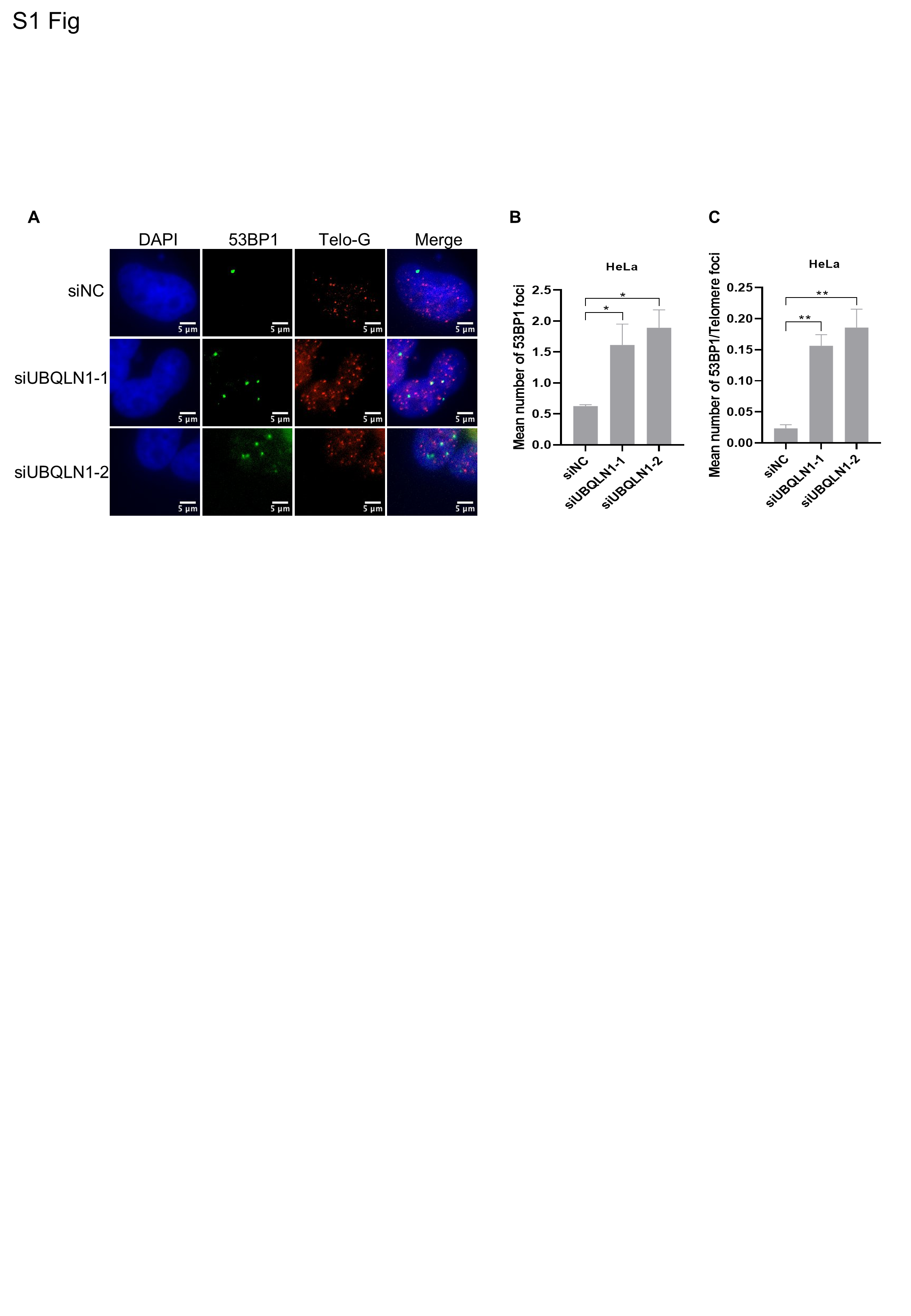

Supplement: S1 Fig — (A) 53BP1 foci increased in UBQLN1 depleted HeLa cells. Cells were transfected with indicated siRNAs for 72 h and IF was performed. Scale bars, 5μm. (B-C) Quantification of (A). Cells contain more than one 53BP1 foci were calculated. Total (B) or telomere localized (C) 53BP1 foci were counted respectively. All values are means ± SEM of more than three independent experiments (* P<0.05, ** P<0.01, *** P<0.001, ****P<0.0001). (TIF) [file pgen.1010856.s001.TIF]

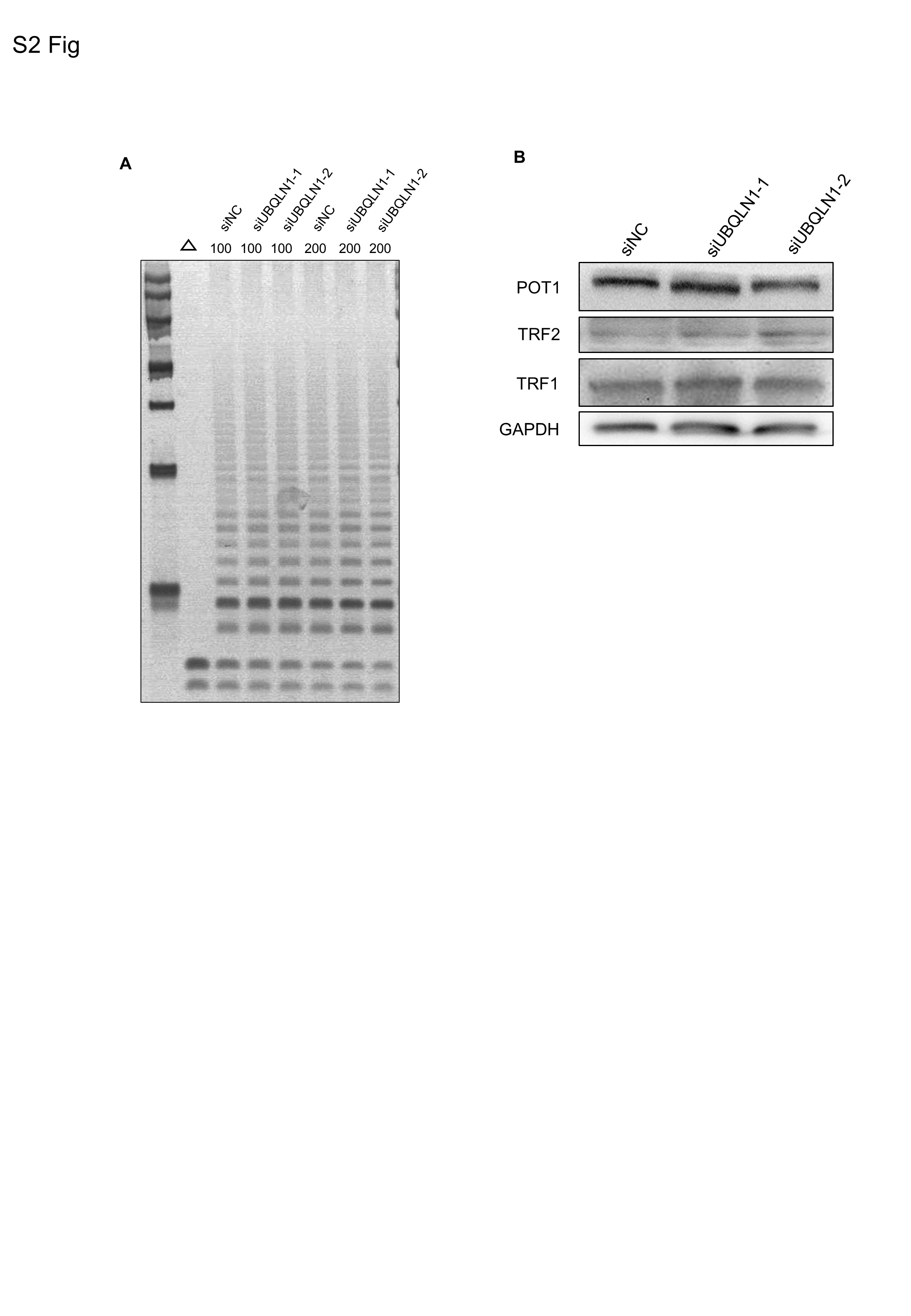

Supplement: S2 Fig — (A) TRAP assay for telomerase activity detection in UBQLN1 depleted cells. (B) Protein levels in UBQLN1 knockdown cells. Immunoblot analysis of indicated proteins in HeLa cells at 72 h after siRNA transfection. (TIF) [file pgen.1010856.s002.TIF]

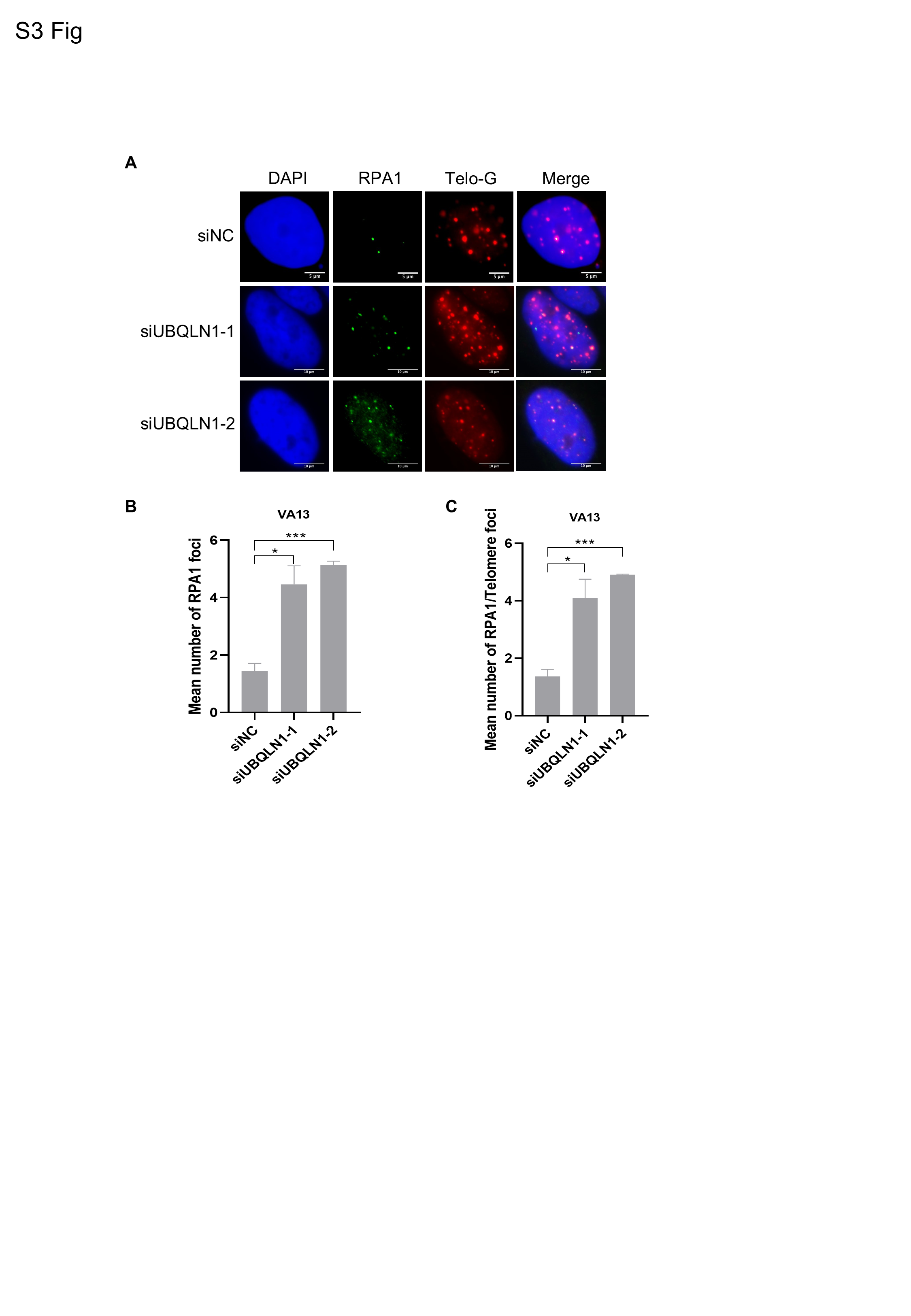

Supplement: S3 Fig — (A) RPA1 foci increased in UBQLN1 depleted VA13 cells. Cells were transfected with indicated siRNAs for 72 h and IF was performed. Scale bars, 5μm. (B-C) Quantification of (A). Cells contain more than one RPA1 foci were calculated. Total (B) or telomere localized (C) RPA1 foci were counted respectively. All values are means ± SEM of more than three independent experiments (* P<0.05, ** P<0.01, *** P<0.001, ****P<0.0001). (TIF) [file pgen.1010856.s003.TIF]

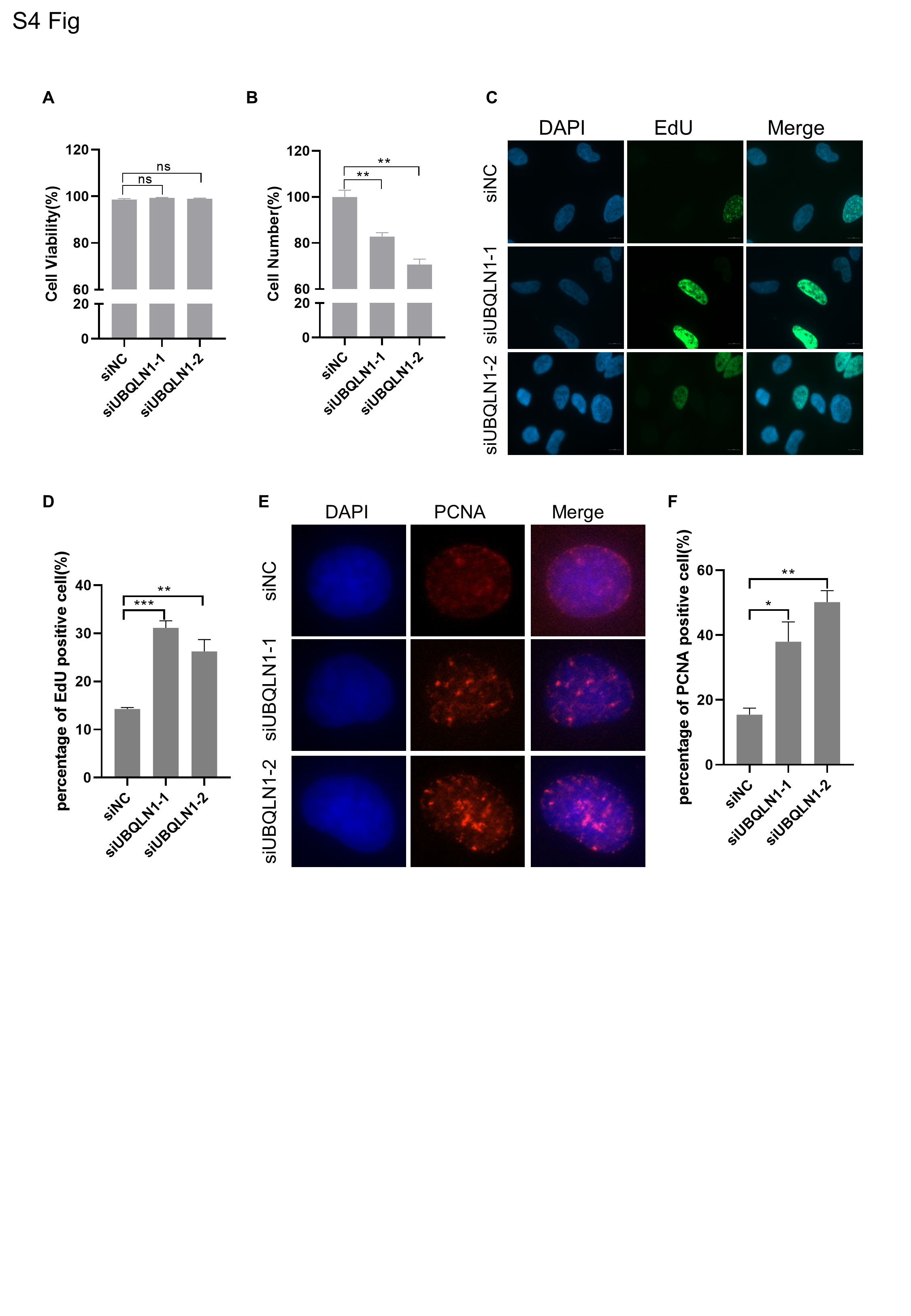

Supplement: S4 Fig — (A) Cell viability assay after UBQLN1 knockdown. Hoechst stained all cells; PI stained death cells. (B) Relative cell number was detected by CCK8 assay. (C) UBQLN1 depletion increases EdU positive cells. HeLa cells were transfected with siRNAs for 72 h and treated with EdU for 15 min. Scale bars, 5 μm. (D) Quantification of (C). The percentage of EdU positive cells were calculated. (E) UBQLN1 depletion increases PCNA positive cells. Hela cells were transfected with siRNAs for 72 h and IF was performed. Scale bars, 5 μm. (F) Quantification of (E). The percentage of PCNA positive cells were calculated. All values are means ± SEM of more than three independent experiments (* P<0.05, ** P<0.01, *** P<0.001, ****P<0.0001). (TIF) [file pgen.1010856.s004.TIF]

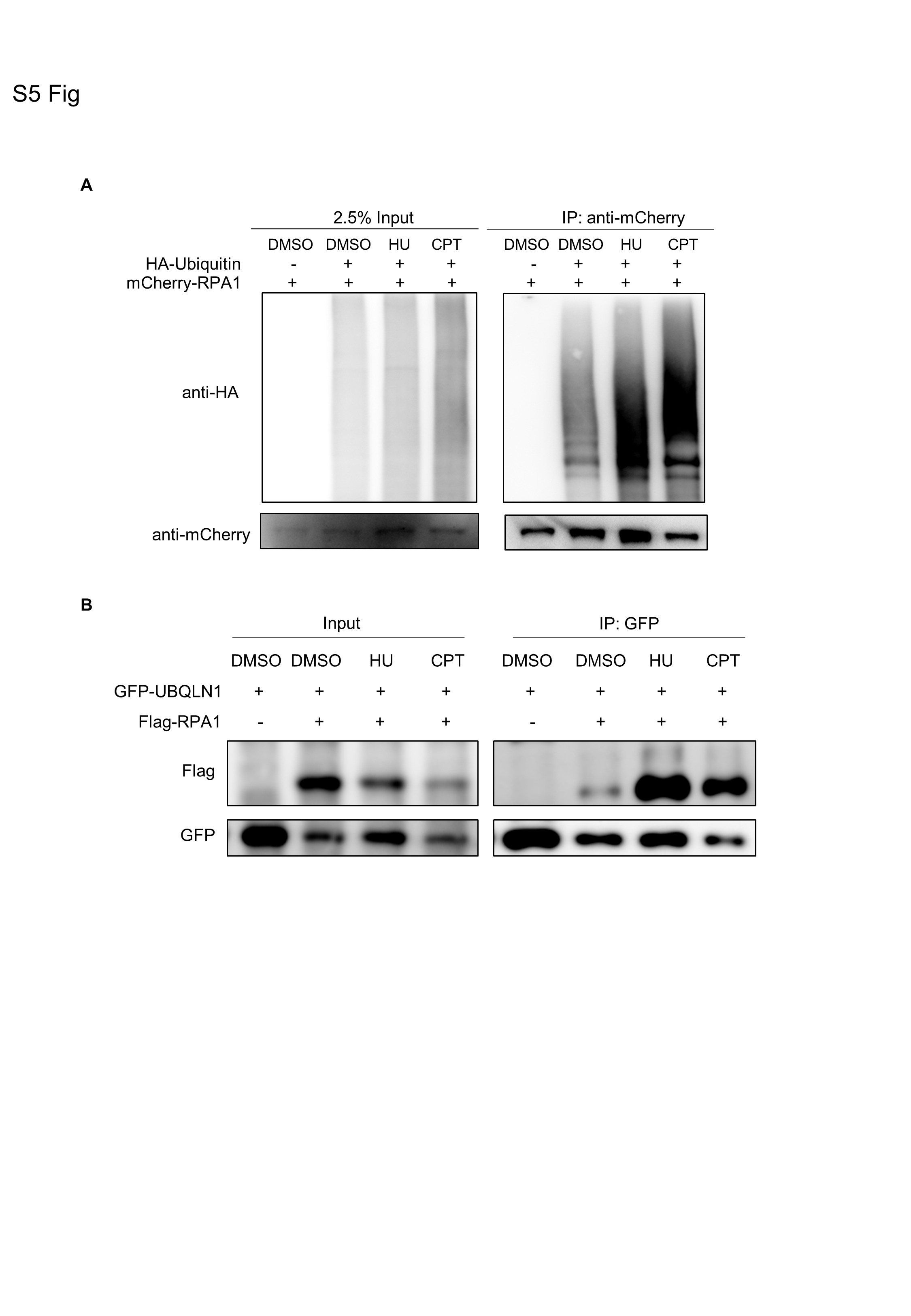

Supplement: S5 Fig — (A) 293T cells were transfected with plasmids expressing HA-Ubiquitin and mCherry-RPA1 or vector for 72 h, and treated with HU (1 mM), CPT (100 nM) or DMSO during the last 12 h. IP-western was performed with indicated antibodies (n = 3). (B) 293T cells were transfected with plasmids expressing HA-Ubiquitin, Flag-RPA1, GFP-UBQLN1 or vector for 72 h, and treated with DMSO, HU (1 mM) or CPT (100 nM) during the last 12 h. Co-IP and western were performed with indicated antibodies (n = 3). (TIF) [file pgen.1010856.s005.TIF]

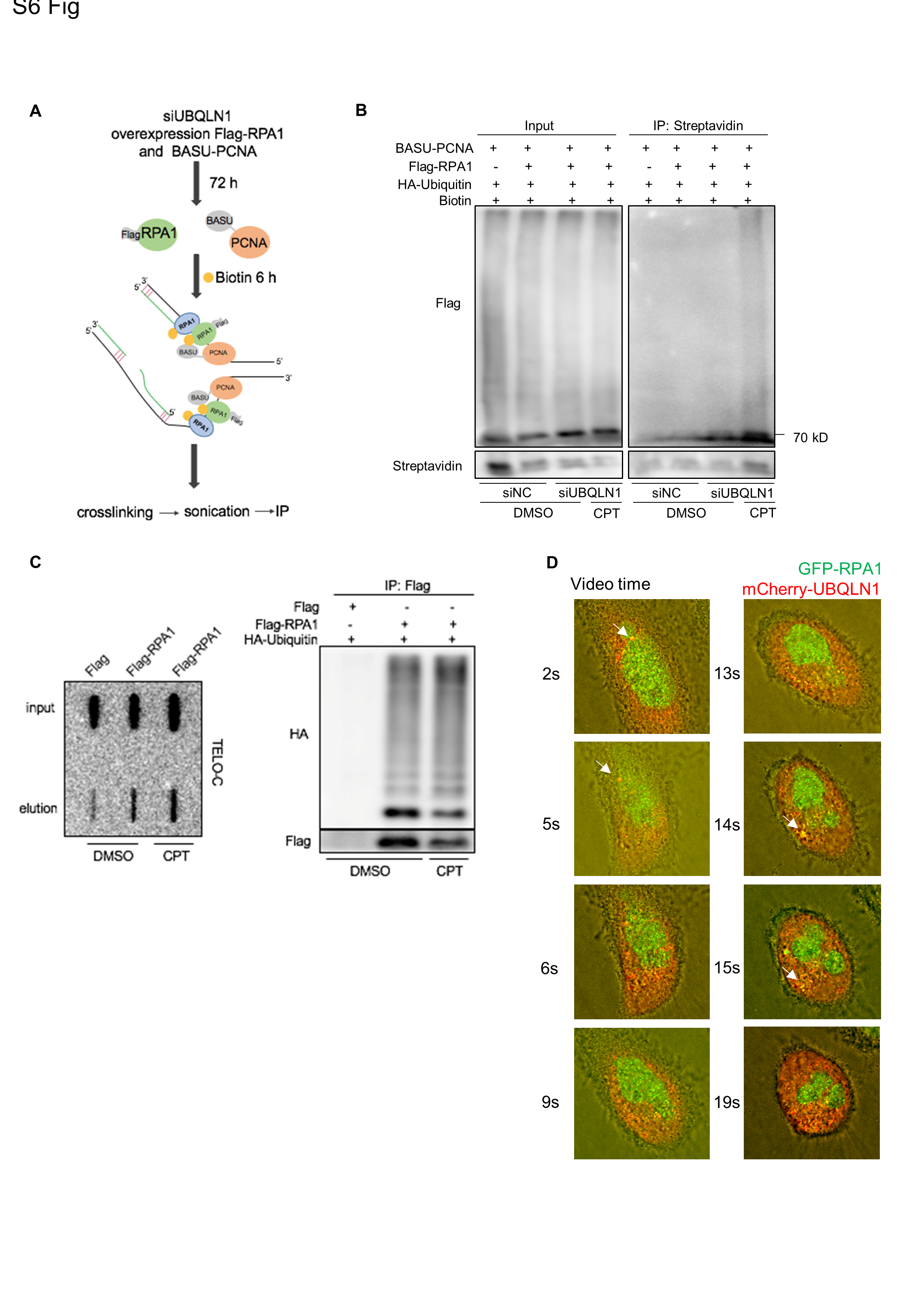

Supplement: S6 Fig — (A) Experimental schematic diagram of B. (B) RPA1 retained at replication fork after UBQLN1 knockdown. 293T cells were transfected with siRNAs and plasmids expressing Flag-RPA1, BASU-PCNA and HA-Ubiquitin for 72h, and then labeled with biotin for 6h. After crosslinking and sonication, Co-IP and western were performed with indicated antibodies. (C) RPA1 ChIP. Half of the precipitate was detected by Western blot using ubiquitin antibody, and the other half was detected by Southern blot using telomeric probe. (D) HeLa cells were transfected with GFP-RPA1 and mCherry-UBQLN1 and synchronizated at S phase as indicated in Fig 5A. The live cell was photographed using live cell imaging technology for 6 hours (n = 1). (TIF) [file pgen.1010856.s006.TIF]
